# Chemoproteomics-based profiling reveals potential antimalarial mechanism of Celastrol by disrupting spermidine and protein synthesis

**DOI:** 10.1186/s12964-023-01409-5

**Published:** 2024-02-20

**Authors:** Peng Gao, Jianyou Wang, Huan Tang, Huanhuan Pang, Jiemei Liu, Chen Wang, Fei Xia, Honglin Chen, Liting Xu, Junzhe Zhang, Lixia Yuan, Guang Han, Jigang Wang, Gang Liu

**Affiliations:** 1 Department of rehabilitation medicine, Shunde Hospital, Southern Medical University, Foshan, 528300 China; 2 State Key Laboratory of Antiviral Drugs, School of Pharmacy, Henan University, Kaifeng, 475004 China; 3 State Key Laboratory for Quality Ensurance and Sustainable Use of Dao-di Herbs, Artemisinin Research Center, and Institute of Chinese Materia Medica, China Academy of Chinese Medical Sciences, Beijing, 100700 China; 4 School of Traditional Chinese Medicine and School of Pharmaceutical Sciences, Southern Medical University, Guangzhou, 510515 China; 5 Guangdong Provincial Key Laboratory of Chinese Medicine Pharmaceutics, Southern Medical University, Guangzhou, 510515 China; 6 Department of Pulmonary and Critical Care Medicine, Shenzhen Institute of Respiratory Diseases, and Shenzhen Clinical Research Centre for Geriatrics, Shenzhen People’s Hospital; First Affiliated Hospital of Southern University of Science and Technology; Second Clinical Medical College of Jinan University, Shenzhen, 518020 China

**Keywords:** Celastrol, Antimalarial, Spermidine, Protein synthesis

## Abstract

**Background:**

Malaria remains a global health burden, and the emergence and increasing spread of drug resistance to current antimalarials poses a major challenge to malaria control. There is an urgent need to find new drugs or strategies to alleviate this predicament. Celastrol (Cel) is an extensively studied natural bioactive compound that has shown potentially promising antimalarial activity, but its antimalarial mechanism remains largely elusive.

**Methods:**

We first established the *Plasmodium berghei* ANKA-infected C57BL/6 mouse model and systematically evaluated the antimalarial effects of Cel in conjunction with in vitro culture of *Plasmodium falciparum*. The potential antimalarial targets of Cel were then identified using a Cel activity probe based on the activity-based protein profiling (ABPP) technology. Subsequently, the antimalarial mechanism was analyzed by integrating with proteomics and transcriptomics. The binding of Cel to the identified key target proteins was verified by a series of biochemical experiments and functional assays.

**Results:**

The results of the pharmacodynamic assay showed that Cel has favorable antimalarial activity both in vivo and in vitro. The ABPP-based target profiling showed that Cel can bind to a number of proteins in the parasite. Among the 31 identified potential target proteins of Cel, *Pf*Spdsyn and *Pf*EGF1-α were verified to be two critical target proteins, suggesting the role of Cel in interfering with the de novo synthesis of spermidine and proteins of the parasite, thus exerting its antimalarial effects.

**Conclusions:**

In conclusion, this study reports for the first time the potential antimalarial targets and mechanism of action of Cel using the ABPP strategy. Our work not only support the expansion of Cel as a potential antimalarial agent or adjuvant, but also establishes the necessary theoretical basis for the development of potential antimalarial drugs with pentacyclic triterpenoid structures, as represented by Cel.

Video Abstract

**Supplementary Information:**

The online version contains supplementary material available at 10.1186/s12964-023-01409-5.

## Background

Malaria is a lethal infectious disease that seriously threatens the health of approximately half of the world’s population, with an estimated 619,000 people worldwide dying from malaria infection in 2021 [1]. In recent decades, the widespread use of artemisinin-based drugs has greatly accelerated the process of malaria elimination. However, the increasing reports of resistance to artemisinin (ART) and ART-based combination therapies (ACTs) in recent years have caused great concern and have also undoubtedly increased the burden of malaria control efforts [2, 3]. Therefore, the development of new antimalarials with potential to alleviate the current predicament is highly desirable. Natural products have long been an important source of novel antimalarials [4, 5]. Triterpenoids and their derivatives have been reported to have potential antimalarial activity and are considered potential lead compounds for novel antimalarials [6].

Celastrol (Cel), a pentacyclic triterpenoid natural product isolated from the traditional Chinese medicine *Tripterygium wilfordii* (“thunder god vine”), is considered one of the five traditional natural medicines most likely to be developed into a modern drug, with anti-inflammatory, immunomodulatory, anti-tumor, and anti-microbial activities [7–9]. Figueiredo et al. found that Cel inhibited the proliferation of *Plasmodium falciparum* (*P. falciparum*) at low uM concentrations in vitro [10]. Subsequently, other studies have shown that Cel has potential antimalarial effects by possibly interfering with the *P. falciparum* fatty acid metabolism and inhibiting parasite Hsp90 [11, 12]. In addition, a recent study showed that Cel can synergize with ART by inhibiting the activity of parasite redox-related enzymes [13].

Despite decades of research, the critical antimalarial targets of Cel and the associated antimalarial mechanisms of action are still not fully understood. To this end, a systematic series of investigations is needed. In the current study, we first evaluated the antimalarial activity of Cel in vivo and in vitro. Subsequently, we identified the antimalarial targets of Cel using the activity-based protein profiling (ABPP) technique based on a Cel activity probe (Cel-P) previously developed by our group [14] and integrated with a combination of proteomics and transcriptomics analysis. A series of biochemical and functional validations were performed to establish the antimalarial mechanism of action of Cel. Overall, our study suggests that Cel can interfere with the process of spermine and protein synthesis in *P. falciparum* by binding to *Pf*Spdsyn and *Pf*EGF1-α, resulting in antimalarial effects. This work deepens our understanding of the antimalarial mechanism of Cel and opens up the possibility of its development as a novel antimalarial agent or adjuvant.

## Methods

### Animal experiments

The in vivo antimalarial activity of Cel was investigated in *P. berghei* ANKA (*Pb*ANKA) infected mice as described previously [15]. The *Pb*ANKA strain was obtained from the Artemisinin Research Center of the China Academy of Chinese Medical Sciences. Infected red blood cells (iRBC) containing *Pb*ANKA parasites were inoculated into C57BL/6 mice by intraperitoneal injection, and blood smears were collected daily to monitor parasite growth by Giemsa staining. When the parasitemia reached 15–20%, blood from infected mice was collected through the eyeball and counted. Finally, 1 × 10^7^ iRBCs diluted with PBS were used for further inoculation. Artesunate (ATS) treatment was used as a positive control. Four days after inoculation, infected mice were treated intraperitoneally with different drugs (Cel, ATS, Cel + ATS) and equal volume of solvent vehicles for 4 days. Parasitemia, body temperature (rectal temeperature) and behaviour were monitored daily. Finally, serum was collected for the determination of aspartate aminotransferase (AST) and alanine aminotransferase (ALT). The liver and spleen were weighed and fixed for histologic examination by hematoxylin-eosin (H&E) staining.

### Parasites culture

The parasite strains of *P. falciparum* 3D7 and Dd2 were obtained from the Artemisinin Research Center of the China Academy of Chinese Medical Sciences. The parasite culture was the same as previously described with minor modifications [16, 17]. Briefly, the parasites were cultured in 50 mL malaria complete medium containing 10.4 g/L RPMI1640, 0.5% albumin, 0.2 g/L gentamycin, 25 μg/mL hypoxanthine, 0.3 g/L L-glutamine, 25 mmol/L HEPES, 2.5 g/L NaHCO_3_ with 2% hematocrit at 37 °C. After several generations of culture, the parasites were synchronized twice in succession using 5% sorbitol. Giemsa staining was used to monitor the parasitemia. Parasitemia was maintained at ~ 8%.

### Antimalarial activity in vitro assay

The 72 h fluorescent SYBR Green I assay was used to measure the antimalarial activity of Cel in vitro [18]. First, 0.05% parasitemia and 2% hematocrit highly synchronized ring stage parasites were seeded into 96-well plates and treated with a series of dilutions of Cel. After incubation, the supernatant was removed and the lysate buffer containing SYBR Green I was added. Fluorescence intensity was measured using an Envision 2105 Multimode Plate Reader (PerkinElmer). The half-maximal inhibitory concentration (IC_50_) and log-concentration-response curves were analyzed using GraphPad Prism 8.

### In situ fluorescence labeling

The fluorescence labeling was performed primarily as previously descripted [14]. Briefly, parasites were seeded in a 6-well plate and co-incubated with Cel-P for 4 h. For the competition assay, parasites were treated with excess Cel for 2 h before Cel-P was added for another 2 h. Infected erythrocytes were then collected and lysed for protein extraction. The protein concentration was measured using the BCA kit (Beyotime, China). An equal amount of protein was used for the click chemistry reaction. The proteins were then precipitated with pre-chilled acetone, redissolved, and separated by SDS-PAGE electrophoresis. The Sapphire Biomolecular Imager (Azure Biosystems) was used to acquire fluorescence gel images and Coomassie Brilliant Blue was used as a loading control stain.

### Intracellular imaging and immunofluorescence

The intracellular fluorescence imaging assay was performed as previously reported [18]. Briefly, parasites were seeded in 24-well plates at 5% parasitemia with 2% hematocrit. The cells were fixed and dropped onto cover slides coated with poly-L-lysine and perforated. The click reaction was then carried out. For confocal imaging of the intracellular probe, the coverslides were inverted on a slide and Leica TCS SP8 SR confocal microscope was used for rapid imaging. For immunofluorescence, the corresponding antibodies were incubated after the click reaction. A Dragonfly 200 Spinning Disk Confocal Microscopy was used for immunofluorescence imaging, and ImageJ software was used for semiquantitative analysis.

### In situ pull-down experiments

To identify the potential antimalarial target proteins of Cel, the pull-down experiments were performed [17]. The method of collecting parasite proteins after treatment is the same as the in situ fluorescence labeling. After quantification, the soluble proteins were used for the click reaction. The proteins were then incubated with Neutravidin beads (Thermo Scientific) for enrichment. Then the proteins were reduced, alkylated and digested with trypsin. After digestion, the peptide-rich supernatant was desalted, spin-dried and labelled with Tandem Mass Tag (TMT) Labeling Reagents. Finally, the enriched peptides were analyzed by the LC-MS/MS. The proteins with fold change (FC) ≥ 1.2 and *P*-value < 0.05 were considered as statistically significant target proteins. Gene ontology (GO) enrichment analysis was performed using Metascape. For Pull-down Western blot analysis, the enriched proteins were released by heating at 95 °C and separated by SDS-PAGE electrophoresis for immunoblotting.

### Expression and purification of recombinant proteins

The proteins were expressed and purified as described previously with slight modifications [18]. The protein sequences of *Pf*Spdsyn with a 29-residue N-terminal deletion [19] and *Pf*EGF1-α were obtained from PlasmoDB database. The genes were cloned into the pET28a vector, transfected into *Escherichia coli* BL21, and cultured in LB medium. IPTG was used to induce the expression of the recombinant protein. After purification, the recombinant proteins were eluted with gradient elution buffer. The protein concentration was measured and stored at − 80 °C for later use.

## Fluorescence labeling of recombinant proteins

Equal amounts of recombinant proteins (20 μg) were incubated with different concentrations of Cel-P at room temperature [20]. In the competition experiment, excess Cel and iodoacetamide (IAA) were added first and then Cel-P or IAA-P was added. The remaining steps were the same as those described above for in situ fluorescence labeling.

## Cellular thermal shift assay

To further investigate the binding ability between Cel and target proteins, cellular thermal shift assay coupled with Western blot (CESTA-WB) was performed as previously described [21]. Briefly, recombinant proteins were treated with Cel or DMSO at room temperature. Next, the proteins were aliquoted into PCR tubes and heated in a thermal cycler (Applied Biosystems, Thermo Scientific) at a range of increasing temperatures (from 45 °C to 61 °C). After high-speed centrifugation at 4 °C, the supernatant was collected and a Western blot experiment was performed.

## UV-visible absorption assay

The UV absorption spectra of Cel were measured at 300-600 nm using a 96-well plate reader (PerkinElmer, USA) [14]. Different concentrations of Cel were diluted and the absorbance was measured. For the absorbance of Cel post binding to target proteins, Cel and recombinant proteins were incubated at room temperature for 1 h, followed by the absorbance measurement.

## Bio-layer interferometry (BLI) assay

The Octet® NTA Biosensor and Octet® Bio-Layer Interferometry (BLI) platform were used to study the binding of Cel to target proteins. First, the recombinant proteins were immobilized on the biosensor and then bound to gradient concentrations of Cel. The baseline time was set to 60s, the molecular association to 90s and the dissociation to 120 s. Octet Analysis Studio (13.0) was used for kinetic analysis.

### Molecular docking simulation

The structure of Cel was downloaded from PubChem (CID:122724), the protein 3D structure of *Pf*Spdsyn from PDB (2PT9) and *Pf*EGF1-α from Alphafold (Q8I0P6). The docking simulation was performed by MOE (2019.0102) [17]. Cysteine in the protein was selected as a potential site and the default method of the software was used for docking. After docking, the optimal result was selected as the potential binding mode.

### Measurement of spermidine level

Spermidine levels were measured using a spermidine ELISA kit (CLOUD-CLONE CORP, CEX053Ge). Parasites were seeded in a 6-well plate and incubated with different concentrations of Cel. Parasites were then collected for the measurement of spermidine according to the manufacturer’s instructions.

### Newly synthesized protein inhibition assay

The labeling of newly synthesized proteins was performed as previously reported [20, 22]. First, parasites were washed with methionine (Met)-free 1640 medium and then incubated in Met-free 1640 medium for 30 minutes. After incubation, 50 μmol/L Click-iT™ AHA (Thermo Fisher Scientific, C10102) and different concentrations of Cel were added, and cycloheximide (CHX) was used as a control. The parasite was harvested for protein extraction and the click reaction was performed to conjugate TAMRA-alkyne or biotin-alkyne. The SDS-PAGE electrophoresis and the pull-down assay steps were the same as the described above.

### Proteomics analysis

Parasites were treated with DMSO or Cel for 6 h. Infected erythrocytes were collected and the proteins were extracted. The same amount of protein was taken for reduction with dithiothreitol (DTT) and reaction with IAA. The proteins were then digested with trypsin overnight at 37 °C [23]. After digestion, the peptides were desalted and spin-drying for LC-MS/MS analysis. Proteins with *P-*value < 0.05 and fold change ≥1.5 were considered as differentially expressed proteins. Metascape was used for GO enrichment analysis.

## RNA-sequencing analysis

The RNA-sequencing (RNA-seq) analysis was performed as previously described [24]. The NEBNext Ultra RNA Library Prep Kit for Illumina (NEB, USA) was used to construct the sequencing library [20]. After treatment with DMSO or Cel, total RNA was extracted from the parasites, followed by mRNA purification using poly-T oligo-attached magnetic beads. PCR experiments were performed and the sequencing results were collected on the Illumina platform (Novogene, CN). Three independent reproducible experiments were carried out for each group. *P-*value < 0.05 and log_2_FC (fold change) ≥ 0.5 were considered differentially expressed genes and ESeq2 (V1.10.0) R package was used to analyse the differential gene expression.

## Results

### Antimalarial activity of Celastrol against *Pb*ANKA infection in mice

**Fig. 1:**
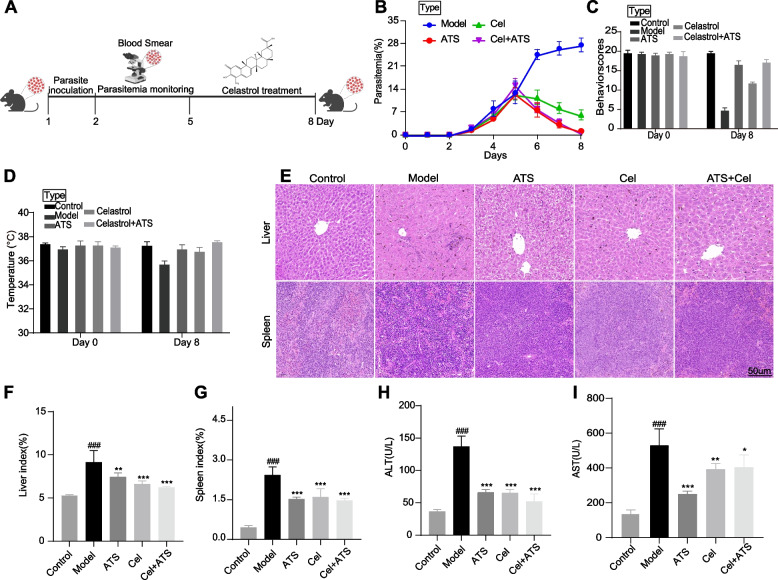
Celastrol shows potential antimalarial effects in vivo in *Pb*ANKA infected C57BL/6 mice. **A** The scheme of animal modeling and drug handling. C57BL/6 mice were divided into five groups (Control; model; ATS treatment; Cel treatment; ATS + Cel treatment). **B** Daily parasitemia was monitored by Giemsa staining. **C-D** The behavioral observations and body temperature measurements on the first day and last day. **E** The histopathological observations of liver and spleen using H&E staining (scale bar = 50 μm). **F-G** The organ index of liver and spleen in each group after treatment. **H-I** The levels of serum ALT and AST in each group after treatment. All data were presented as mean ± standard error of the mean (SEM), ^###^*P <* 0.001 vs control; ^*^P < 0.05, ^**^P < 0.01, ^***^P < 0.001 vs model. ATS, artesunate; Cel, Celastrol

First, we evaluated the in vivo antimalarial effects of Celastrol (Cel) in *Pb*ANKA-infected mice as shown in Fig. 1A [25]. The results showed that there was a significant reduction in blood parasitemia in infected mice after Cel treatment alone, with a trend similar to that of the artesunate-treated group (Fig. 1B). And the trend in the group treated with both artesunate and Cel was consistent with that of artesunate alone (Fig. 1B). Similar trends were observed in behavioral changes as well as changes in apparent body temperature (Fig.1C-D). During malaria infection, the two most important immune organs, liver and spleen, swell and undergo varying degrees of inflammatory damage. Histopathologic evaluation showed that both artesunate and Cel treatment significantly reduced organ enlargement, decreased inflammatory cell infiltration, and mitigated tissue damage, but did not restore them to a normal state (Fig. 1E-G). Notably, Cel treatment seemed to ameliorate liver injury better, suggesting that Cel may have a potential protective effect on the liver [26, 27]. The changes in serum levels of alanine transaminase (ALT) and aspartate transaminase (AST) after drug treatment also suggest that Cel may significantly ameliorate malaria-induced liver injury (Fig. 1H-I).

### Profiling the protein targets of Celastrol by ABPP

**Fig. 2:**
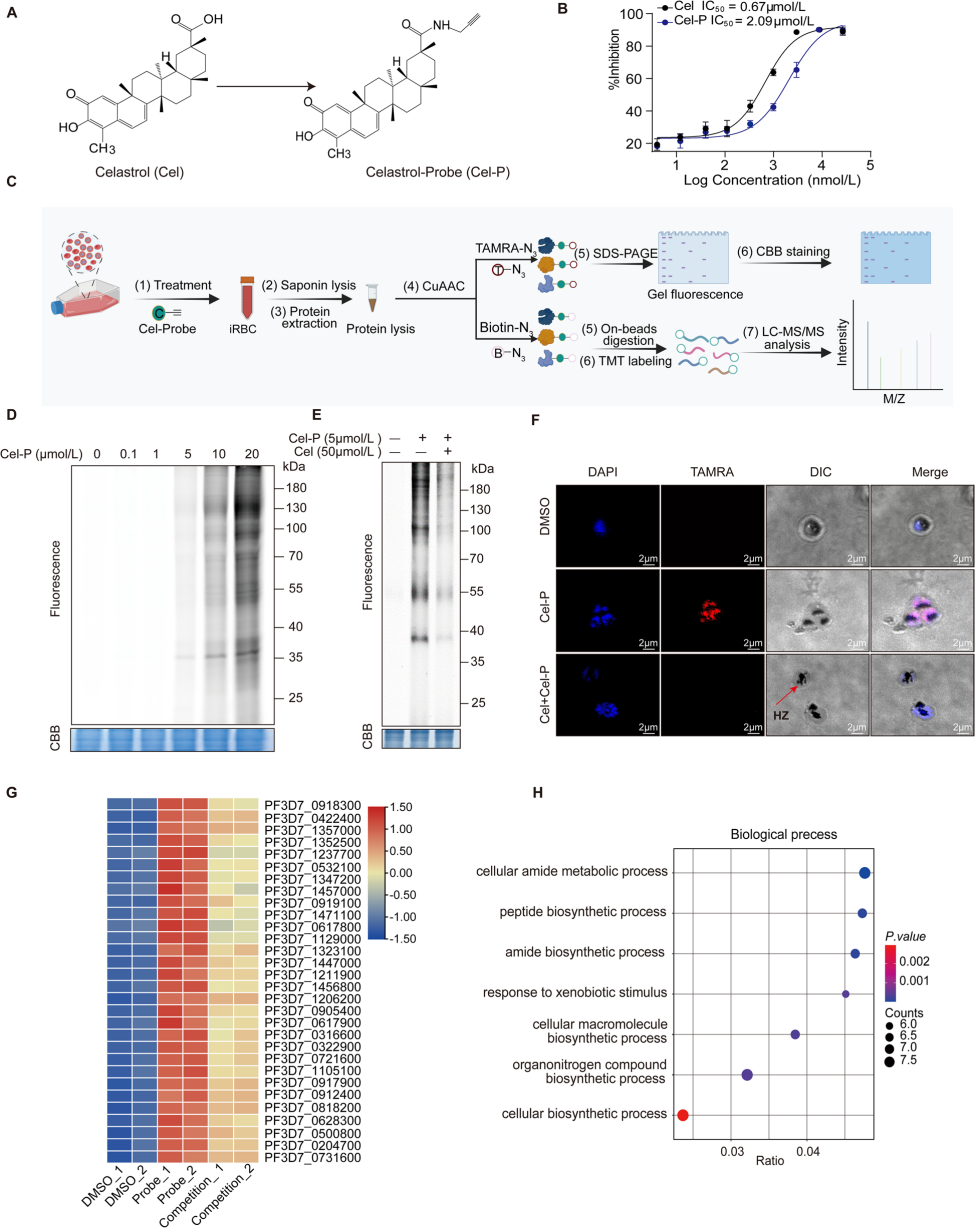
Identification of Cel potential antimalarial targets in *P. falciparum* based on the ABPP strategy. **A** The structure of Cel and Cel-P. **B** The antimalarial activity of Cel and Cel-P against *P. falciparum* 3D7 strain. **C** The workflow of ABPP for the profiling of Cel target proteins. **D** In situ fluorescence labeling of Cel-P in parasite proteins. **E** In situ competition experiment of Cel-P by Cel. **F** Confocal imaging showed the distribution of Cel-P in parasites with or without excess Cel (scale bar = 1 μm). **G** Heatmap representation of the target proteins identified by the Cel-P. **H** Gene ontology (GO) enrichment analysis for all potential targets of Cel-P. CBB, Coomassie brilliant blue; RBC, red blood cells; HZ, hemozoin; TAMRA, carboxy tetramethyl rhodamine

After confirming the antimalarial activity of Cel, we investigated its binding targets in *P. falciparum* using a Cel activity probe (Cel-P) based on ABPP technology (Fig. 2A) [28]. First, we measured the antimalarial activity of the Cel-P against *P. falciparum* 3D7 and Dd2 strains in vitro and compared it with that of Cel. The results showed that the probe retained similar parasiticidal activity as Cel in the low uM concentration range as shown in Fig. 2B and S1. Cel-P was then used to label the parasite proteins by in situ fluorescence labeling assay (Fig. 2C). The results showed that the parasite proteins could be labeled by Cel-P in a dose-dependent manner (Fig. 2D). More importantly, pre-incubation of the excess Cel significantly attenuated the fluorescent labeling, suggesting that Cel and Cel-P target the same proteins in the parasite (Fig. 2E). Furthermore, the live imaging results also showed that Cel-P could quickly distribute inside the parasites and could be outcompeted by excess Cel, which was consistent with the fluorescence labeling in the gel (Fig. 2F). The promising antimalarial activity and target-binding specificity of the Cel-P rasies the possibility of its further usage to identify antimalarial targets of Cel.

Therefore, Cel-P was used to capture and enrich the target proteins based on pull-down experiments (Fig. 2C). Subsequently, the enriched proteins were subjected to quantitative proteomic analysis using high-resolution tandem mass spectrometry (LC-MS/MS) coupled with TMT labeling, and a total of 31 potential targets were identified based on stringent screening criteria (Fig. 2G, Table. S1). Gene ontology (GO) analysis revealed that these potential target proteins were mainly associated with the metabolic and biosynthetic processes of the parasite (Fig. 2H).

### Integrated proteomics and transcriptomics analysis of *P. falciparum* after Celastrol treatment

**Fig. 3:**
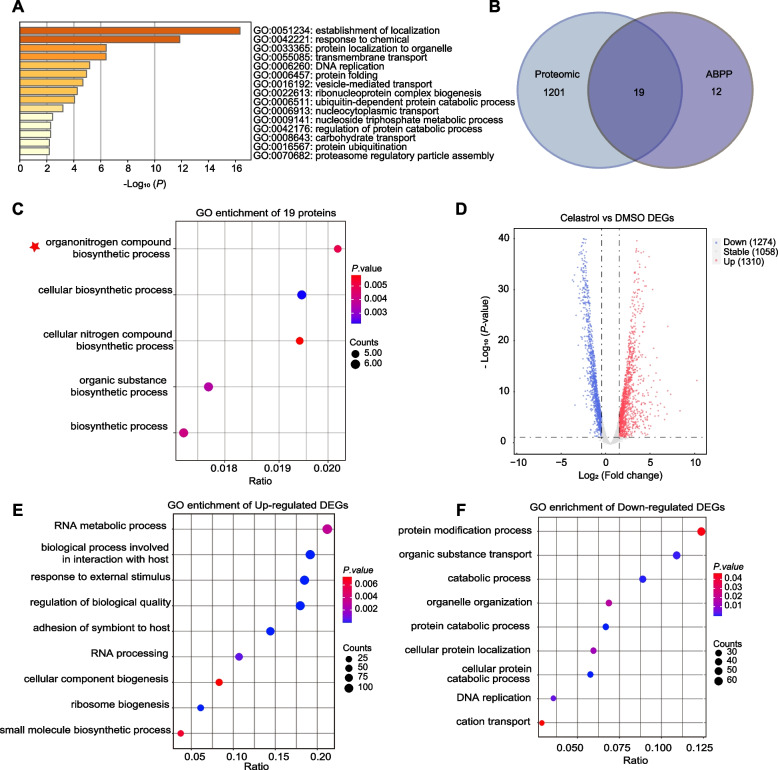
Integrated transcriptomics and proteomics analysis. **A** GO enrichment analysis for the differential proteins in proteomics after Cel treatment. **B** Venn diagrams showing the overlap of differential proteins in proteomics with the target proteins identified by Cel-P. **C** GO enrichment analysis for the overlapped proteins from (**B**). **D** Volcano plot showing the gene expression after Cel treatment. **E-F** GO enrichment analysis of the differential genes in transcriptomics

Next, we investigated the changes at the proteome level of *P. falciparum* after Cel treatment by proteomics analysis. The results showed significant changes in the expression of 1220 proteins in the whole proteome after Cel treatment (Fig. S2), and GO enrichment analysis showed that these proteins were mainly involved in various physiological processes such as protein synthesis (Fig. 3A). We then examined the changes in protein expression of the potential targets identified by Cel-P and found 19 targets with significant changes in protein expression (Fig. 3B), while the results of GO analysis indicated that these proteins are involved in several important physiological processes (Fig. 3C). From the significantly enriched biological processes, we found that biosynthetic processes are the most likely processes for Cel targeting. To further validate the targeting of Cel in these processes, in the following sections, we performed a series of verification experiments on two important and representative proteins of interest, *Pf*Spdsyn and *Pf*EGF1-α, which have been shown to play important roles in parasite growth and are considered to be promising drug targets.

We should note that, we also investigated the changes at the transcriptome level of *P. falciparum* after Cel treatment by transcriptomics analysis. The results showed that significant regulation occurred at the transcriptome level, with 1310 genes being up-regulated and 1274 genes being down-regulated (Fig. 3D), involving multiple physiological processes (Fig. 3E-F). This further reflects that Cel may have potential multi-targeted antimalarial properties.

### Celastrol inhibits the spermidine synthesis in *P. falciparum*

Polyamines are essential and ubiquitous for cell proliferation and differentiation. They interact with various anionic macromolecules (DNA, RNA, protein, etc.) to regulate a variety of physiological activities, such as influencing the DNA, RNA, and protein synthesis and affecting ion channels [29]. Inhibition of polyamine synthesis has been suggested as a promising antimalarial approach, and enzymes involved in polyamine biosynthesis are considered promising drug targets [30]. Das Gupta et al. showed that polyamine levels in *P. falciparum* correlate with its growth stage, and that spermidine is the most abundant polyamine at all times [31]. In *P. falciparum*, spermidine is synthesized via the transfer of an aminopropyl moiety to putrescine by spermidine synthase (*Pf*Spdsyn, PF3D7_1129000), and since *P. falciparum* lacks polyamine interconversion, the activity of *Pf*Spdsyn determines the level of spermidine in *P. falciparum* [32–34].

**Fig. 4:**
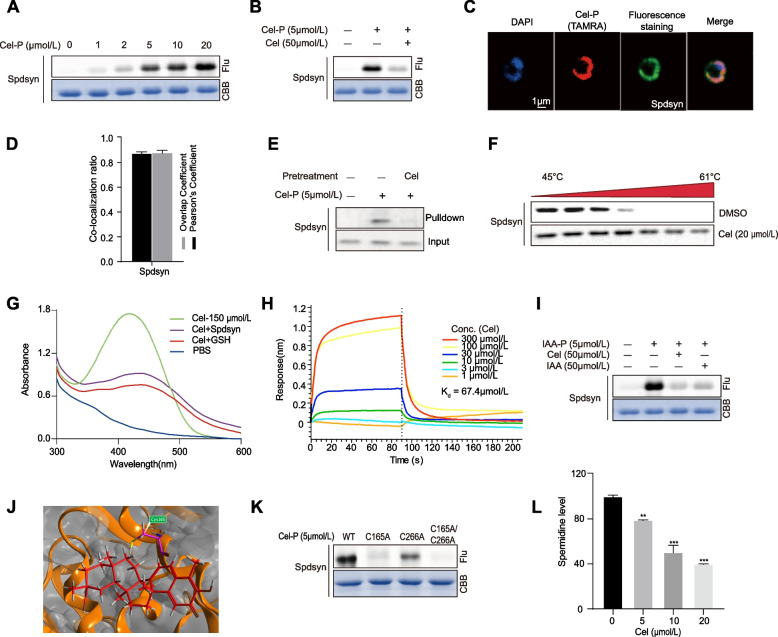
Validation of Cel binding to *Pf*Spdsyn (PF3D7_1129,000). **A** Fluorescence labeling of recombinant *Pf*Spdsyn protein with Cel-P in a dose-dependent manner. **B** Competition fluorescence labeling of Cel-P with excess Cel. **C-D** Immunofluorescence staining of co-localization of Cel-P with *Pf*Spdsyn and quantitative analysis of colocalization. **E** Validation of the binding of Cel-P to *Pf*Spdsyn using pull-down Western blotting. **F** Validation of the binding of Cel-P to *Pf*Spdsyn using cellular thermal shift assay. **G** The UV absorbance spectra of Cel after incubation with *Pf*Spdsyn. **H** The measurement of binding affinity of Cel with *Pf*Spdsyn using the Bio-layer interferometry (BLI) assay. **I** Competition fluorescence labeling of IAA-P with Cel and IAA. (**J**) Docking simulation of Cel binding to Cys165 of *Pf*Spdsyn. **K** Fluorescence labeling of Cel-P on the wild-type (WT), single-site mutants (C165A, C266A), and double-site mutant (C165A/C266A) of *Pf*Spdsyn. **L** The spermidine level of parasites after treatment with different concentrations of Cel. All data were presented as mean ± standard error of the mean (SEM), **P < 0.01, ***P < 0.001

As shown above, *Pf*Spdsyn was identified as a potential antimalarial target of Cel. We expressed and purified the recombinant *Pf*Spdsyn protein in vitro and performed a series of experiments to validate its interaction with Cel [19]. The fluorescence labeling experiments showed that Cel-P binds the *P. falciparum* protein in a dose-dependent manner and can be specifically competed away by excess Cel (Fig. 4A-B). Similar results were also observed in subsequent live cell immunofluorescence experiments as well as in pull-down western blotting experiments (Fig. 4C-E). Meanwhile, the cellular thermal shift assay coupled with Western blotting (CETSA-WB) experiment showed that Cel could significantly increase the thermal stability of *Pf*Spdsyn protein compared to the DMSO control group (Fig. 4F). In addition, the UV absorption was significantly attenuated after incubation of Cel to *Pf*Spdsyn protein (Fig. S3, Fig. 4)G [14]. In addition, the Bio-layer interferometry (BLI) assay also demonstrated that the interaction of Cel with *Pf*Spdsyn protein (Fig. 4H). Previous studies have shown that Cel can form a covalent bond to cysteine residues of proteins through the Michael addition reaction [35]. As expected, in the competitive labeling experiments, Cel significantly attenuated the labeling of IAA-alkynyl probe (IAA-P), a commonly used cysteine-targeting probe (Fig. 4I). Molecular docking simulation identified cysteines 165 and 266 as potential binding sites for Cel on *Pf*Spdsyn proteins, which was validated by fluorescence labeling experiments on the corresponding single-site and double-site mutants (Fig. 4J-K). What’s more, we also measured the spermidine levels in parasites after Cel treatment, and it can be seen that spermidine levels decreased in a dose-dependent manner (Fig. 4L). Taken together, the above experimental results suggest that Cel may interfere with the spermidine production in *P. falciparum* by binding to *Pf*Spdsyn and exert its antimalarial effect.

### Celastrol inhibits the de novo protein synthesis in *P. falciparum*

During the asexual intraerythrocytic life cycle, *P. falciparum* exhibits extremely high levels of transcription and translation, accompanied by massive protein synthesis for rapid growth and proliferation [36], which has long been a focus of attention in antimalarial drug development and has been extensively studied [37, 38]. *P. falciparum* elongation factor 1-α (*Pf*EGF1-α, PF3D7_1357000) is a high-abundance protein primarily involved in the translation process, which binds to ribosomes and plays an indispensable role in *P. falciparum* protein synthesis [36, 39]. In addition, *Pf*EGF1-α is also reported to be involved in other important physiological processes such as cytoskeletal rearrangements and protein degradation [36].

**Fig. 5:**
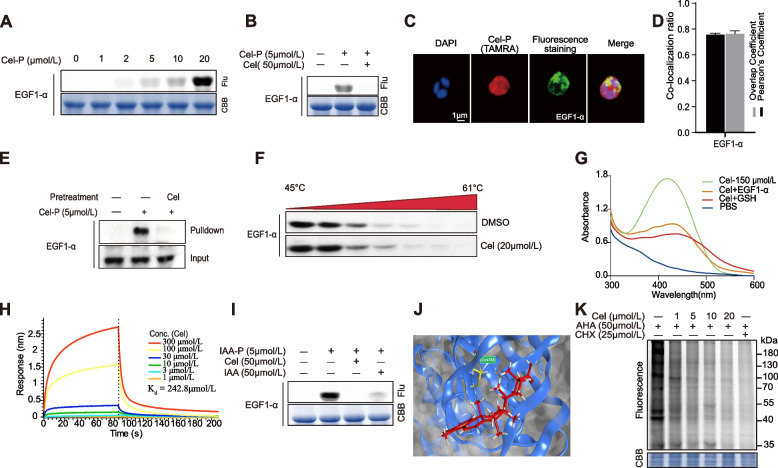
Validation of Cel binding to *Pf*EGF1-α (PF3D7_1357000). **A-J** Similar validations as performed in Fig. 4(A-J). **K** The decrease in fluorescence of AHA-labeling after the treatment with Cel. CHX (cycloheximide) serves as a positive control for the inhibition of protein synthesis

In the present study, *Pf*EGF1-α was also identified as a potential target of Cel. We then performed a series of experiments to validate that Cel can specifically bind to *Pf*EGF1-α by reacting with cysteine residues, similar to the experiments performed for the *Pf*Spdsyn protein (Fig. 5A-J). We note that, several ribosomal proteins were also identified as potential antimalarial targets of Cel (Table S1), further predicting that Cel may affect parasite protein synthesis, similar to the finding in a previous study of Cel on tumour cells [40]. We then detected the changes in de novo protein synthesis in *P. falciparum* after Cel treatment using a non-radioactive L-methionine analog AHA based on the ABPP method [20, 22, 41, 42]. The results showed that Cel could indeed interfere with the protein synthesis of *P. falciparum* (Fig. 5K)*.* Meanwhile, we also identified and analyzed the proteins whose synthesis was inhibited by LC-MS/MS as mentioned above. The results showed that the synthesis of 171 proteins was inhibited by Cel, and further GO analysis indicated that these proteins were mainly related to several critical physiological processes, such as transmembrane transport, carbohydrate transport, and others (Fig. S4).

## Discussion

Pharmacological treatment is the mainstay of malaria control. In recent decades, the widespread use of artemisinin-based drugs has greatly slowed the global malaria epidemic, but with the gradual emergence of resistance to artemisinin and artemisinin-based combination therapies [43, 44]. The antimalarial situation has become more critical, increasing the need and search for new antimalarial drugs [45]. Natural products have always been an important source for antimalarial drug development and play a pivotal role in the antimalarial process. Cel, as one of the most widely studied natural bioactive compound, has shown great potential in anti-rheumatoid arthritis [46], anti-tumour [47] and neuronal protection [48], and has also been reported to have favourable in vitro antimalarial activity, but little has been reported on the mechanism of action, which has hindered its applications in malaria treatment.

**Fig. 6:**
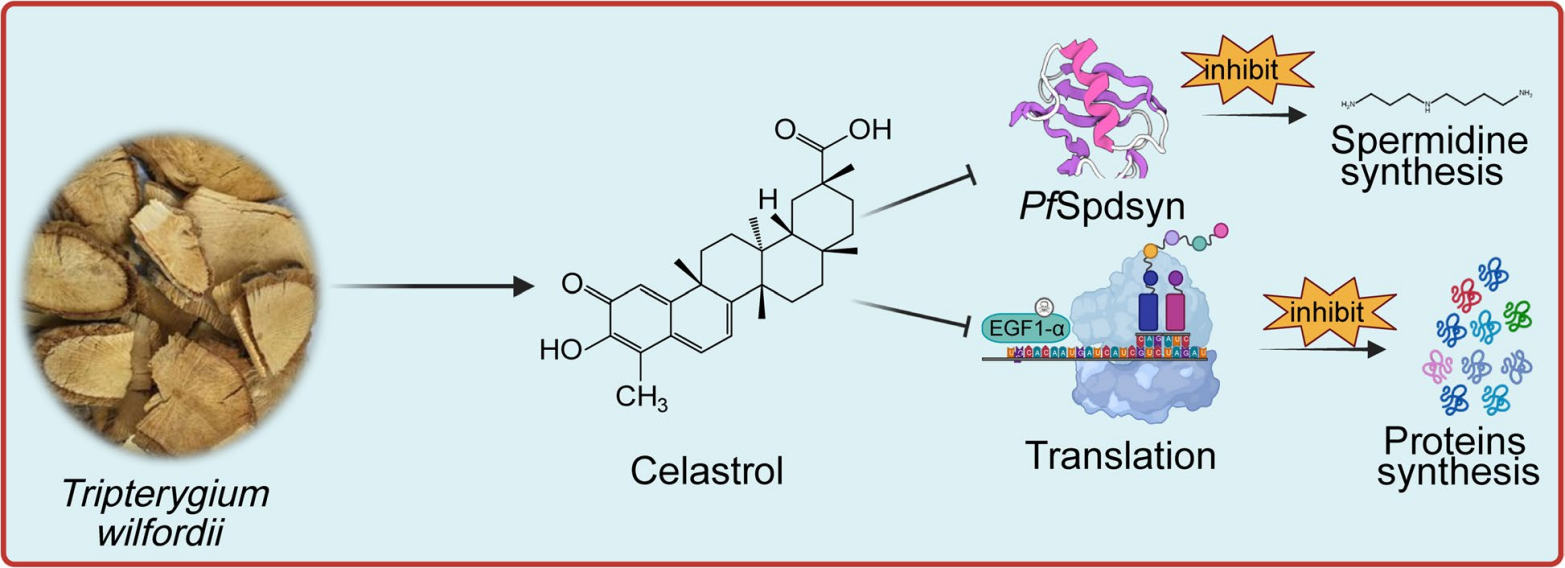
The potential antimalarial mechanism of Celastrol

In this work, we systematically evaluated the antimalarial activity of Cel by establishing animal models and in vitro parasite cultures, identified its targets using ABPP technology, and performed a series of validations [49]. We found that Cel can exert antimalarial activity by binding to *Pf*Spdsyn and *Pf*EGF1-α proteins, thereby interfering with the process of spermidine and protein synthesis in *P. falciparum* (Fig. 6). Targeting polyamine and protein synthesis has been proposed as an attractive antimalarial therapeutic strategy, and the associated proteins are considered promising drug targets [50, 51]. In addition, our analyses suggest that Cel also appears to affect other biological processes, but further work may be needed to explore these in more depth and comprehensively. What’s more, we have showed that Cel also has excellent antimalarial activity against artemisinin-resistant strains (Fig. S5) [52], suggesting that using Cel as a backbone to develop novel antimalarials may alleviate the current artemisinin resistance predicament.

To the best of our knowledge, this is the first report on the identification of antimalarial targets of Cel and associated mechanism of action studies using the ABPP technology, which is of great value for the further exploitation of Cel. Following this work, we will chemically synthesize more Cel-based derivatives and investigate their antimalarial activities, in the hope of finding new antimalarial drugs with excellent pharmacological and physicochemical properties. We believe that this would be a very useful strategy for the development of new antimalarial drugs.

## Conclusions

Overall, this work has deepened our understanding of Celastrol in antimalarials by investigating its potential antimalarial targets and mechanisms of action, while opening up the possibility of further developing Celastrol as a novel antimalarial drug or adjuvant. More importantly, this work has also provided a good start and established the necessary theoretical basis for the development of potential antimalarial drugs with pentacyclic triterpenoid structures, as represented by Celastrol. Last but not least, our work also provides an impetus to explore other natural compounds with potential antimalarial activity.

### Supplementary Information

**Additional file 1: Fig. S1.** The antimalarial activity of Cel and Cel-P against *P. falciparum* Dd2 strain. **Fig. S2.** Heatmap representation of the proteome after Celastrol treatment. **Fig. S3.** The absorbance spectra of increasing concentration Celastrol. **Fig. S4. A** Heatmap representation of the decreased expression of parasite proteins after Cel treatment. **B** GO enrichment analysis of the decreased expression proteins. **Fig. S5.** The antimalarial activity of Cel against artemisinin-sensitive (*P. falciparum* 3D7) and artemisinin-resistant strains (*P. falciparum* 6320). **Fig. S6.** Raw data of all gel images and Western blots.

**Additional file 2: Table S1.** Identified target proteins list by Cel-P.

## Data Availability

The datasets used and/or analyzed during the present study are available from the corresponding author on reasonable request.

## Abbreviations

ABPP: Activity-based protein profiling
AHA: L-azidohomoalanine
ALT: Aminotransferase
ART: Artemisinin
AST: Aminotransferase
ATS: Artesunate
BLI: Bio-Layer Interferometry
CBB: Coomassie brilliant blue
Cel: Celastrol
Cel-P: Celastrol activity probe
CESTA: Cellular thermal shift assays
CHX: Cycloheximide
DTT: Dithiothreitol
GO: Gene ontology
HZ: Hemozoin
IAA: Iodoacetamide
IPTG: Isopropylthio-β-galactoside
LB medium: Luria–Bertani medium
Met: Methionine
*P. falciparum*: *Plasmodium falciparum*
*Pf*EGF1-α: *Plasmodium falciparum* elongation factor 1-alpha
*Pf*Spdsyn: *Plasmodium falciparum* spermidine synthase
RBC: Red blood cells
RNA-seq: RNA-sequencing
TAMRA: Carboxy tetramethyl rhodamine
