# Supplementary material for: Chemoproteomics-based profiling reveals potential antimalarial mechanism of Celastrol by disrupting spermidine and protein synthesis
Source: Cell Commun Signal. 2024 Feb 20;22:139. doi: 10.1186/s12964-023-01409-5 (PMC10877925; doi:10.1186/s12964-023-01409-5)
Supplement: Supplementary file 2 — Additional file 1: Fig. S1. The antimalarial activity of Cel and Cel-P against P. falciparum Dd2 strain. Fig. S2. Heatmap representation of the proteome after Celastrol treatment. Fig. S3. The absorbance spectra of increasing concentration Celastrol. Fig. S4. A Heatmap representation of the decreased expression of parasite proteins after Cel treatment. B GO enrichment analysis of the decreased expression proteins. Fig. S5. The antimalarial activity of Cel against artemisinin-sensitive (P. falciparum 3D7) and artemisinin-resistant strains (P. falciparum 6320). Fig. S6. Raw data of all gel images and Western blots. [file 12964_2023_1409_MOESM1_ESM.pdf]

Supplementary Material

Chemoproteomics-based profiling reveals potential antimalarial mechanism of Celastrol by disrupting spermidine and protein synthesis

Peng Gao[1]†, Jianyou Wang[2]†, Huan Tang[3]†, Huanhuan Pang[3]†, Jiemei Liu[1], Chen Wang[3], Fei Xia[3], Honglin Chen[2], Liting Xu[3], Junzhe Zhang[3], Lixia Yuan[4,5*], Guang Han[2*], Jigang Wang[2,3,6*], Gang Liu[1*]

[1] Department of rehabilitation medicine, Shunde Hospital, Southern Medical University, Foshan, 528300, China

[2] School of Pharmacy, Henan University, Kaifeng 475004, China

[3] State Key Laboratory for Quality Ensurance and Sustainable Use of Dao-di Herbs, Artemisinin Research Center, and Institute of Chinese Materia Medica, China Academy of Chinese Medical Sciences, Beijing 100700, China

[4] School of Traditional Chinese Medicine and School of Pharmaceutical Sciences, Southern Medical University, Guangzhou 510515, China

[5] Guangdong Provincial Key Laboratory of Chinese Medicine Pharmaceutics, Southern Medical University, Guangzhou 510515, China

[6.]Shenzhen Institute of Respiratory Disease, Shenzhen People's Hospital （First Affiliated Hospital of South University of Science and Technology of China and Second Affiliated Hospital of Jinan University, Shenzhen 518020, China

[*]Address correspondence to:

lg2781@smu.edu.cn (G.L.); jgwang@icmm.ac.cn (J.W.); hang@henu.edu.cn (G.H.); cnylxtcm@smu.edu.cn (L.Y.).

†These authors contributed equally to this work.

Supplementary Figures

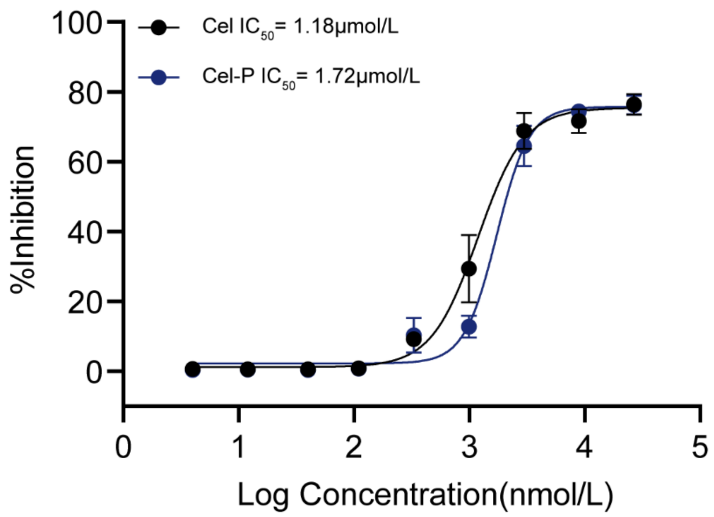

Figure S1 The antimalarial activity of Cel and Cel-P against *P. falciparum* Dd2 strain. All data were based on three independent biological replicates at least, and shown as mean ± standard error of the mean (SEM).

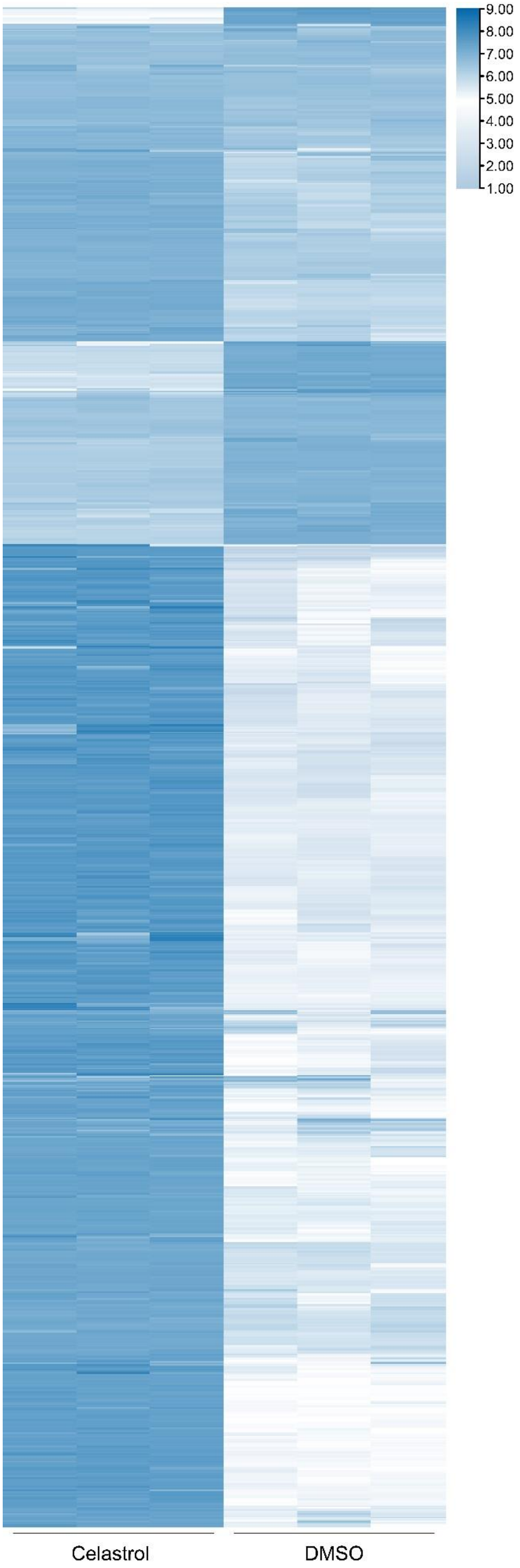

Figure S2 Heatmap representation of the proteome after Celastrol treatment.

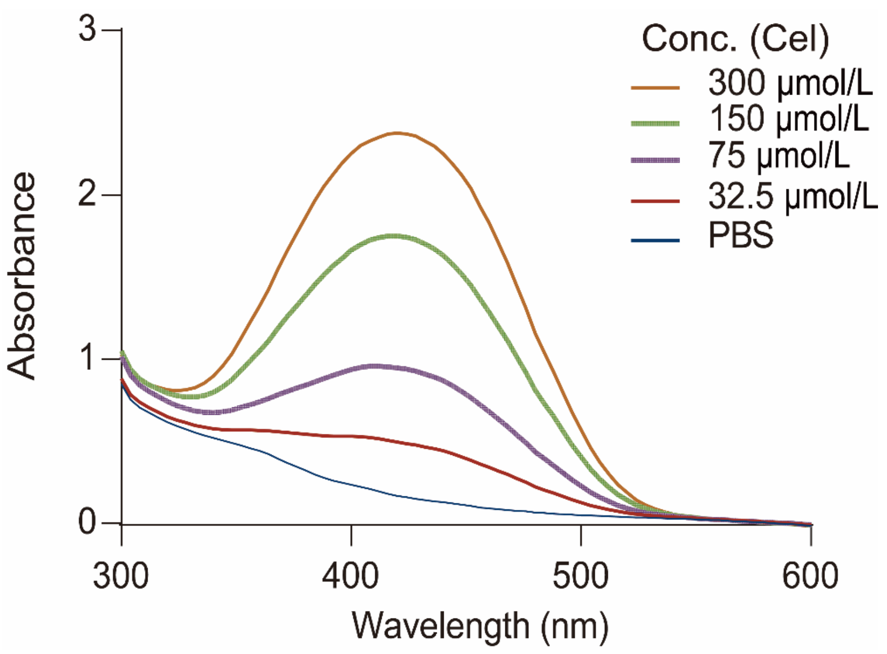

Figure S3 The UV absorbance spectra of increasing concentration Celastrol.

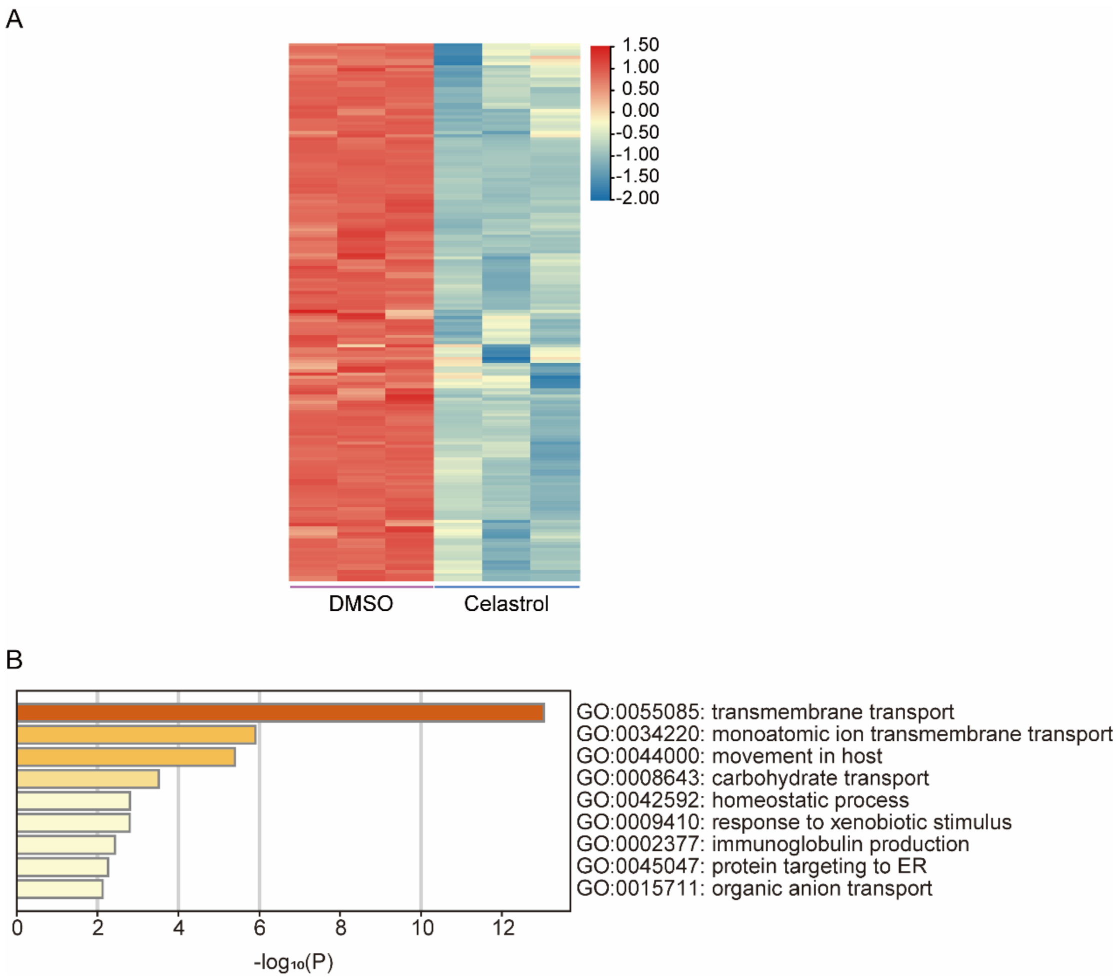

Figure S4 (A) Heatmap representation of the decreased expression of parasite proteins after Cel treatment. (B) GO enrichment analysis of the decreased expression proteins.

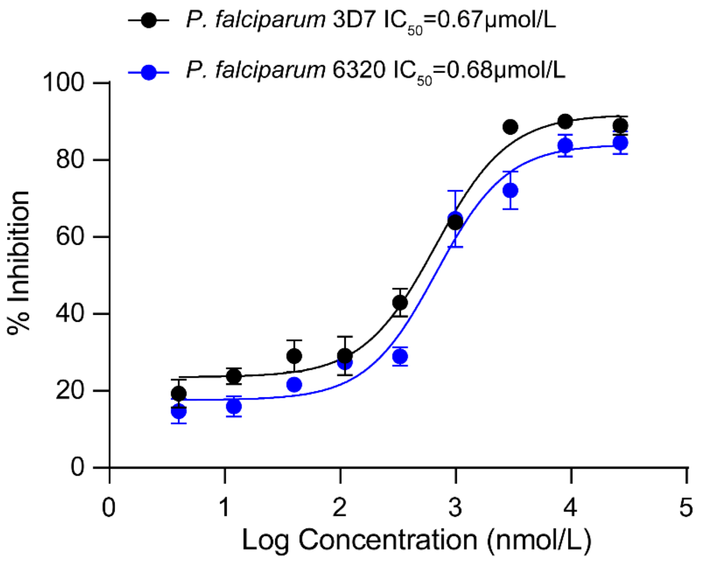

Figure S5 Determination of antimalaria activity of Celastrol against artemisinin-sensitive (*P. falciparum* 3D7) and artemisinin -resistant strains (*P. falciparum* 6320). All data were based on three independent biological replicates at least, and shown as mean ± standard error of the mean (SEM).

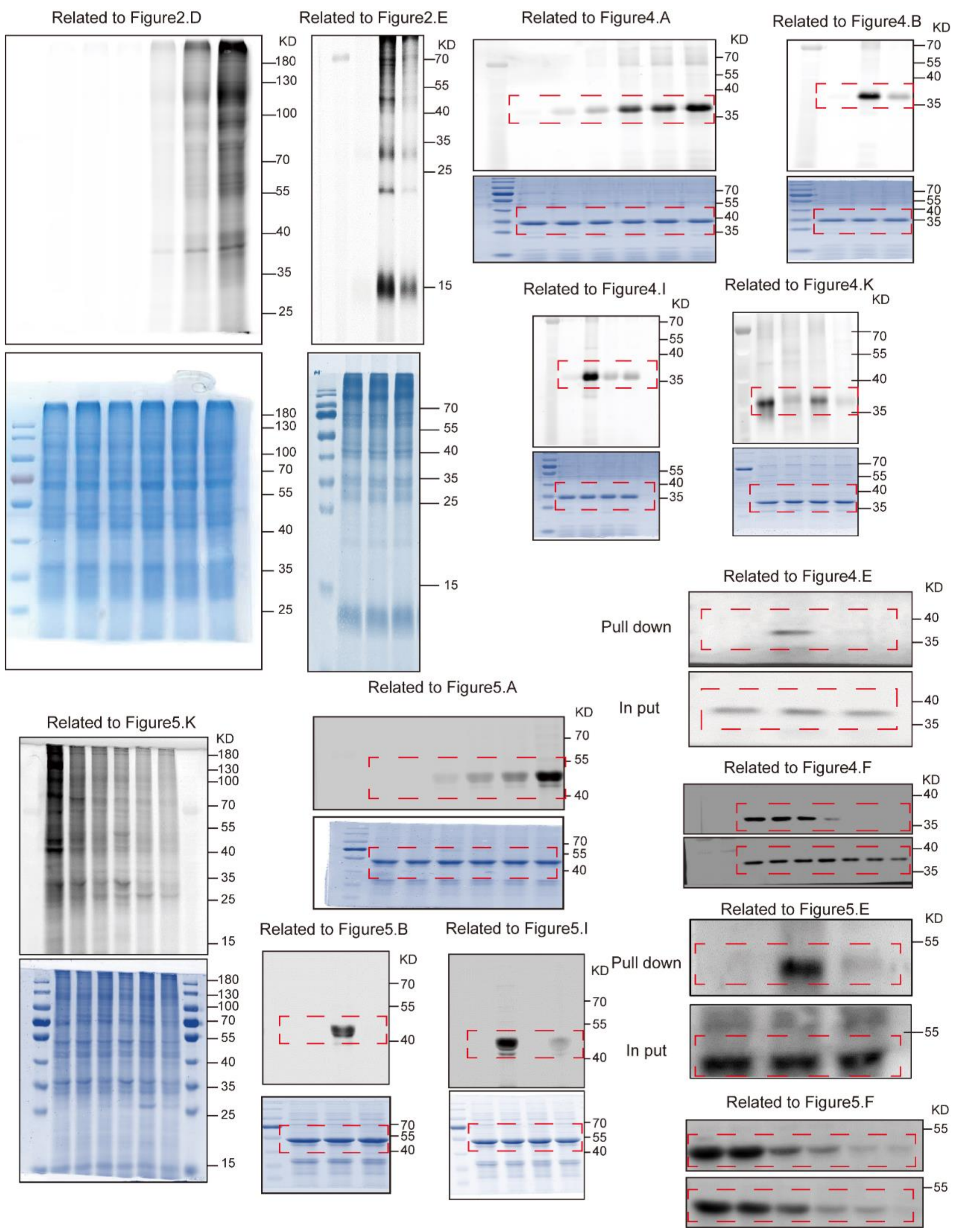

Figure S6 Raw data of gel images and western blots.
