# Supplementary material for: Chemoproteomics-based profiling reveals potential antimalarial mechanism of Celastrol by disrupting spermidine and protein synthesis
Source: Cell Commun Signal. 2024 Feb 20;22:139. doi: 10.1186/s12964-023-01409-5 (PMC10877925; doi:10.1186/s12964-023-01409-5)
Supplement: Supplementary file 3 — Additional file 2: Table S1. Identified target proteins list by Cel-P. [file 12964_2023_1409_MOESM2_ESM.pdf]

Supplementary Table S1

Table S1 Identified target proteins list by Cel-P

**NO.**	**Accession**	**Description**	**MW (kDa)**	**Log_2 FC_D***	**Log_2 FC_C****
1	PF3D7_0918300	eukaryotic translation initiation factor 3 subunit F, putative	36.8	0.86	3.55
2	PF3D7_0422400	40S ribosomal protein S19	19.7	0.43	3.24
3	PF3D7_1357000	elongation factor 1-alpha	48.9	0.3	2.87
4	PF3D7_1352500	thioredoxin-related protein, putative	24	0.72	3.8
5	PF3D7_1237700	conserved protein, unknown function	23.6	1.1	3.05
6	PF3D7_0532100	early transcribed membrane protein 5	19	0.8	3.12
7	PF3D7_1347200	nucleoside transporter 1	47.6	0.87	3.32
8	PF3D7_1457000	signal peptide peptidase	47.5	0.97	3.02
9	PF3D7_0919100	DnaJ protein, putative	43.2	0.46	2.18
10	PF3D7_1471100	exported protein 2	33.4	0.89	3.06
11	PF3D7_1132800	aquaglyceroporin	28.3	0.59	0.72
12	PF3D7_0617800	histone H2A	14.1	1.34	3.87
13	PF3D7_1129000	spermidine synthase	36.6	1.06	3.76
14	PF3D7_1323100	60S ribosomal protein L6, putative	21.6	0.55	3.47
15	PF3D7_1447000	40S ribosomal protein S5	29.9	0.64	3.4
16	PF3D7_1211900	non-SERCA-type Ca2+ -transporting P-ATPase	140.2	0.62	3.5
17	PF3D7_1456800	V-type H(+)-translocating pyrophosphatase, putative	76.4	0.83	3.6
18	PF3D7_1206200	eukaryotic translation initiation factor 3 subunit C, putative	115.9	0.29	2.61
19	PF3D7_0905400	high molecular weight rhoptry protein 3	104.8	0.51	3.3
20	PF3D7_0617900	histone H3 variant	15.4	0.77	3.63
21	PF3D7_0316600	formate-nitrite transporter	34.4	0.71	3.83
22	PF3D7_0322900	40S ribosomal protein S3A, putative	30	0.68	4.18
23	PF3D7_0721600	40S ribosomal protein S5, putative	21.8	0.64	3.4
24	PF3D7_1105100	histone H2B	13.1	0.7	3.49
25	PF3D7_0917900	heat shock protein 70	72.3	0.45	3.27
26	PF3D7_0912400	alkaline phosphatase, putative	52.7	0.31	3.21
27	PF3D7_0818200	14-3-3 protein	30.2	0.37	3.42
28	PF3D7_0628300	choline/ethanolaminephosphotransferase,	45.1	0.6	4.12
29	PF3D7_0500800	mature parasite-infected erythrocyte surface antigen	168.2	0.33	3.07
30	PF3D7_0204700	hexose transporter	56.4	0.54	3.68
31	PF3D7_0731600	acyl-CoA synthetase	93.2	0.33	3.18

*FC_D: Fold change (Probe/DMSO)

**FC_C:Fold change (Probe/Competition)
